# Effectiveness of Helminth Therapy in the Prevention of Allograft Rejection: A Systematic Review of Allogeneic Transplantation

**DOI:** 10.3389/fimmu.2020.01604

**Published:** 2020-08-07

**Authors:** Michelle Kiss, Heather Burns, Sheila Donnelly, Wayne J. Hawthorne

**Affiliations:** ^1^Centre for Transplant and Renal Research, The Westmead Institute for Medical Research, Westmead Hospital, Sydney, NSW, Australia; ^2^University of Sydney, Sydney, NSW, Australia; ^3^School of Life Sciences, University of Technology Sydney, Sydney, NSW, Australia; ^4^Department of Surgery, Western Clinical School, University of Sydney, Sydney, NSW, Australia

**Keywords:** transplantation, allograft survival, helminths, helminth therapy, immune regulation

## Abstract

**Background:** The unique immunomodulatory capacity of helminth parasites has been investigated as a novel strategy in the prevention of allograft rejection after transplantation. This review was conducted to fully evaluate the specific effects of helminth therapy on allograft survival reported in published studies of animal models of allogeneic transplantation.

**Method:** Following PRISMA protocol guidelines, a literature search was conducted using PubMed, MEDLINE via OvidSP, along with additional manual searches of selected reference lists. Publications describing helminth intervention within allograft transplantation models were screened for relevance to eligibility criteria. Primary and secondary outcomes were extracted using standardized data collection tables. The SYRCLE risk of bias assessment tool was used for quality assessment. Due to heterogeneity of study designs, meta-analysis could not be performed; rather outcomes are presented as a narrative synthesis with concept mapping. This review was registered in PROSPERO with ID: CRD42018097175.

**Results:** The literature search generated 1,443 publications, which after screening for relevance to the eligibility criteria yielded 15 publications for qualitative analysis. All 15 publications reported improvement to allograft survival as a result of helminth therapy. This prolonged allograft survival was not significantly different when helminth-derived products were used compared to live infection. However, the extent of positive impact on allograft survival was noted to be dependent on study design factors, such as the chronicity of the live helminth infection, allograft type and the species/genus of helminth selected.

**Conclusion:** Both live and product-based helminth therapy have potential applications as novel immune regulators or adjuncts for the prevention of allograft rejection. However, there were differences in efficacy between different worms and preparations of worm-derived products. Therefore, further studies are required to determine the most appropriate worm for a specific allograft, to elucidate the optimal dose and route of administration, and to better understand the modulation of immune responses that can mediate tolerance.

## Background

Parasitic worms (helminths) are a highly divergent group of macro-pathogens consisting of two major phyla: the Nematoda (or roundworms) and the Platyhelminthes (also known as flatworms), with the latter consisting of both trematodes (flukes) and cestodes (tapeworms) (1). Reflecting this diversity, the course of infection in mammalian hosts varies greatly for different helminths. For example, infection with *Trichinella spiralis* occurs through ingestion of meat that is contaminated with the larval stage of the parasite. In the case of schistosomiasis, infection occurs after the parasite cercaria is released from its intermediate snail host and burrows through the skin of its mammalian host. Larvae migrate through the lung and heart before they mature in the liver. Although infection with *Nippostrongylus brasiliensis* also occurs through subcutaneous infection, unlike the schistosomes that ultimately inhabit the mesenteric blood vessels, *N. brasiliensis* larvae exit the circulation into the lungs and are coughed up and swallowed, eventually residing in the intestine of its chosen mammalian hosts. Despite such diversity in the morphology and life cycles of individual helminths, this group of parasites induces a stereotypical Th2 immune response, characterized by the presence of cytokines IL-4, IL-5, IL-13, and IL-10, antibody isotypes IgG1, IgG4, and IgE, and expanded populations of eosinophils, mast cells, basophils and M2 macrophages (2).

It has been proposed that this specific profile of immune response has evolved as a mechanism to regulate the extensive tissue damage caused by these macro-pathogens as they migrate through the host, resulting in a dynamic co-evolution between the host and the parasite (2, 3). Indeed, the Th2 immune response during parasite infection appears to be initiated by pro-inflammatory cytokines released from damaged or disrupted epithelial cells, with the resultant activation of M2 macrophages and Th2 cells in particular contributing to the host mechanisms of rapid tissue repair through the deposition of extracellular matrix proteins (2, 4).

In contrast to micro-pathogens, such as bacteria, protozoa, and viruses, most helminths do not replicate in their mammalian hosts. Instead, the infective stages must establish infection and then grow to sexual maturity, producing eggs or live offspring for transmission to the next host. Therefore, maintaining host fitness is in the best interest of the parasite. This is supported by the observation that in addition to host signals of damage, a number of parasite-secreted compounds also modulate host immune responses to ensure a positive outcome for both host and parasite (4, 5).

Frequently, helminth parasites establish chronic infections and if untreated can persist for decades in the tissue of their hosts. During these long-term infections, it has been noted that the dynamics of the T cell response changes over time. To prevent excessive tissue repair, which may result in fibrosis-related pathology, the Th2 response often declines during chronic infection and is replaced with the development of regulatory pathways, including the differentiation of regulatory T cells (Tregs). This transition, actively initiated by both host and parasite signals, ensures the fine-tuning of the parasite-driven immune response to restrain pathological outcomes (5, 6).

This potent modulation of host immunity by helminths has additional unintended consequences, resulting in the regulation of a variety of inflammatory conditions caused by a dysregulated immune response, such as rheumatoid arthritis, multiple sclerosis, ulcerative colitis, type 1 and 2 diabetes, and sepsis (7–10). In both natural and experimental helminth infections, there is a clear inverse relationship between the presence of parasites and the progression of inflammatory disease (11–13). These observations have led to the proposition of helminth therapy. However, due to the challenges of manufacturing a consistent batch of live parasites from mammalian hosts that is acceptable to current regulatory standards, combined with concern regarding the pathology of parasite infection, there has not been broad clinical support for the use of live infections as a therapeutic strategy. As such, the focus of this field of research has shifted to the characterization of the molecules that are excreted and secreted by helminths to support the pharmacological development of worm-derived immunomodulators as therapeutics or to exploit their mechanism of action to develop small molecule drugs (14–16).

In light of this immunomodulatory capability, many have suggested extending the use of helminth therapy to the prevention of allograft rejection. Successful allograft transplantation is continually plagued by the challenge of both acute and chronic cell mediated allograft rejection (17). This process is mediated through both indirect and direct forms of antigen recognition and is typically regulated through the release of pro-inflammatory Th1 cytokines (17, 18). Current immunosuppressive regimens aim to dampen cell-mediated rejection by targeting various immune cell interactions. Although successful in suppressing allograft rejection, these immunosuppressive agents carry significant toxicities, and their use can lead to increased rates of infection and malignancies and can also be toxic to the transplanted organ (19, 20). Considering this, the potential application of helminths as a natural, novel immune regulatory therapy for allograft rejection represents a therapeutic advantage.

To date, there has been no single comprehensive study on the use of helminth therapy as an adjunct to traditional immunosuppressive therapy. In particular, the analysis of the impacts of study design, the choice of parasite, and importantly, the difference between live and product-based helminth therapy on the survival of allografts has not been determined. Therefore, this systematic review aims to apply these considerations to the qualitative analysis of published studies to better characterize the effects of live and product-based helminth therapy on the survival of allogeneic transplants.

## Methods

This systematic review was conducted according to the provided Preferred Reporting Items for Systematic Reviews and Meta-Analysis guidelines (PRISMA) (21) and registered with PROSPERO (registration number: CRD42018097175).

### Information Sources and Literature Search Strategy

Publications were gathered from database searches of PubMed and Medline via OvidSP. Additional publications were compiled from reference lists of literature reviews and included studies until January 2020. The following search terms were utilized for both databases: (helminth^*^ OR nematode OR trematode OR cestode OR worm^*^ OR “parasitic worm^*^” OR schistosome^*^ OR *trichuris* OR *nippostrongylus* OR fluke OR tapeworm OR hookworm) AND (transplant^*^ OR allograft OR “allograft survival” OR “skin graft”). Publications were screened for duplicates and for relevance to outlined criteria. A secondary reviewer provided resolution to any discrepancies in publication relevance.

### Eligibility Criteria and Study Selection

This review focused on the impact of helminthic therapy on allograft survival. To be eligible, studies were required to investigate any model of allogeneic organ transplantation and consider the following criteria:

#### Interventions

Any form of helminthic therapy, including live and helminth-derived products, in any form or species/genus, dose, and route of administration or duration, in comparison to placebo/control group were considered for review. Helminth derived products could involve any form including native secretions of a parasite, soluble worm extracts from homogenous worms or eggs and purified recombinant single proteins derived from helminths. Placebo/control groups were considered as any treatment not containing helminthic intervention, e.g., saline.

#### Populations

All animal populations were considered for review. There were no exclusions relating to age, species, or weight of animals.

#### Study Design

All forms of study design were included for review, irrespective of sample size.

#### Outcomes

For inclusion, the administration of helminths in any form must have impacted allograft survival. Any studies in which helminth infection occurred but was not implemented as a treatment were excluded.

#### Exclusion

Non-English publications and review publications and papers not available in full paper form were excluded for review. Additionally, *in vitro* and *in silico* studies were not considered.

### Data Items and Data Collection Process

All eligible publications were reviewed, and relevant data items were extracted using a standardized data collection table. Data items provided in the collection table included publication date, helminth genus/species, allograft model, live or product-based therapy, parasite burden, administration route and time, as well as worm life stage and sample size. Data items were organized into groups according to helminth genus type before identifying live or product-based therapeutic design. Within product-based therapeutic designs, studies were further grouped according to product type, such as soluble worm extracts, native secretions of the worm, and purified recombinant proteins. Outcomes and results were assessed in comparison to control, with allograft survival recorded as rejection (days post-transplantation).

### Bias and Quality Assessment

Bias was assessed by two reviewers using an adapted form of the SYRCLE risk of bias assessment tool, a version of the Cochrane RoB tool (22). Items 1, 3, 5, and 6 were removed, as they were unlikely to impact on the outcomes of the publications. Each reviewer assessed bias according to the questions within each item, indicating risk of bias by answering yes, no or unclear to the guided questions. Discrepancies in answers between each reviewer were resolved by discussion with a third reviewer. The percentage of bias present for each publication was determined by reporting the percentage yes, no and unclear answers in a bar graph. Bias was also reported as a quality assessment score and averaged for each item of the assessment tool. Publications that failed to report at least 50% of the items were considered of poor quality and hence excluded from this review.

### Summary Measures and Synthesis of Results

Due to heterogeneity of the publications, meta-analysis was not applicable for this systematic review. Studies are instead presented in narrative synthesis, detailing the effect of helminth therapy on allograft survival. The primary outcomes of graft survival (days post-transplantation) was reported in addition to supplementary data presented in each study, including immunohistochemistry, flow cytometry and serum cytokine detection. Differences in graft survival for each allograft presented, were compared by considering the helminth genus and species, allograft type, and parasite burden utilized. In addition, the effect of live and product-based intervention type was considered for its impact on graft survival. These were then presented as concept maps to visualize the impact of such study design characteristics on the primary outcome.

## Results

### Study Selection

**Figure 1:**
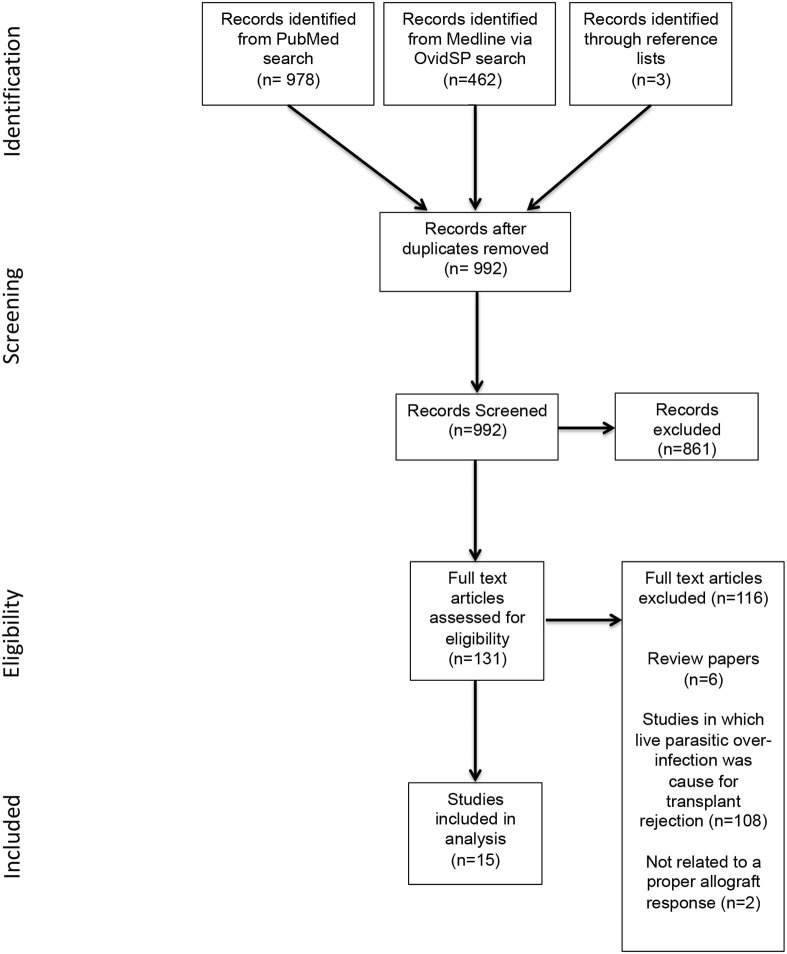
PRISMA flowchart of study selection.

As shown in Figure 1, the initial database and manual reference list search yielded 1,443 publications. Removal of duplicates left 989 publications, of which 861 publications were excluded based on irrelevance. The remaining 131 publications were assessed for eligibility based upon inclusion criteria, resulting in a further 116 being excluded. A total of 15 relevant publications were included for review.

### Study Characteristics

The characteristics of the included studies are summarized in Supplementary Table 1. Studies ranged in publication dates from 1969 to 2016. Sample sizes ranged from 5 to 100, with a total of 339 animals infected with live helminth or helminth-derived products across all allograft transplant studies.

The effects of helminth therapy were investigated within multiple allograft models including skin (*n* = 9) (23–31), heart (*n* = 3) (32–34), kidney (*n* = 1) (35), and liver (*n* = 1) (36). One study investigated both skin and heart allograft models (37). Helminth intervention varied according to species/genus, parasite burden, live or product based and administration route. Five different helminths were utilized as treatment, including *Echinococcus multilocularis* (*n* = 2) (32, 36), *Nippostrongylus brasiliensis* (*n* = 2) (34, 35), *Schistosoma mansoni* (*n* = 2) (24, 33), *Paragonimus westermani* (*n* = 1) (29), *Trichinella spiralis* (*n* = 6) (25–28, 30, 37), and a combination of *Trichinella spiralis* and *Trichinella pseudospiralis* (*n* = 2) (23, 31). Helminth therapy was divided into live (*n* = 9) (24, 26–28, 30, 32, 34, 36, 37) and product-based (*n* = 2) (29, 33) treatment, with four studies investigating the difference between live and product-based intervention (23, 25, 31, 35). Within these publications, product based therapeutics were defined as soluble worm extracts (*n* = 5) (23, 25, 29, 35), native secretions from the worm (*n* = 1) (23), or purified recombinant proteins (*n* = 1) (33). Administration of these therapies was given as intraperitoneal (*n* = 4) (24, 29, 32, 36), oral intubation/probe (*n* = 5) (23, 26–28, 37), and subcutaneous injection (*n* = 3) (33–35). One publication did not clearly define administration (30), whilst two other publications used a combination of oral/intraperitoneal injection (25) or intraperitoneal/subcutaneous injection (31). Each intervention was also altered in accordance with parasite burden.

The primary outcome of all publications was allograft survival. This was described as time until graft rejection (days post-transplant) for control vs. treatment for the vast majority of studies. One publication did not report the time until graft rejection (33). Graft rejection was clearly defined (with specific descriptions for distinct transplant types) as bleeding and shrinking of graft (*n* = 2) (23, 24), necrosis (*n* = 7) (25–28, 30, 31, 36), 80% or more induration and no hair growth (*n* = 2) (29, 37), loss of graft function (*n* = 1) (34) increases to morbidity signs (*n* = 1) (35), or cessation of cardiac beating (*n* = 3) (32, 33, 37).

Whilst the majority of publications primarily reported allograft survival as the only outcome, six publications also reported secondary measures to confirm improved allograft survival. These were reported as a combination of histopathology and immunohistochemistry (*n* = 4) (32, 35–37), flow cytometry (*n* = 6) (32–37), direct cytotoxic T lymphocyte activity (*n* = 1) (34), serum cytokine detection by Luminex or ELISA (*n* = 3) (34, 36, 37), and PCR (*n* = 1) (36). In addition, three of the publications noted that the immune modulation induced by helminths involved T regulatory cells (32, 33, 37).

### Bias and Quality Assessment

**Figure 2:**
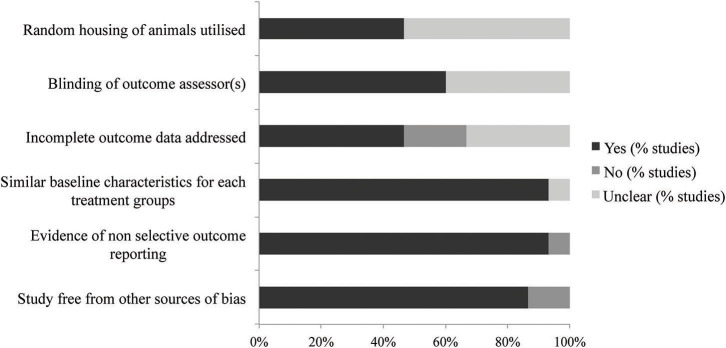
Quality assessment score, averaged per item.

The results of the SYRCLE bias assessment of the 15 publications included in this systematic review are reported in Table 1 and Figure 2. The overall quality of all publications was high, with no studies excluded on this basis. Across all 15 publications, an average of four out of the six chosen items were adequately reported. Three papers reported all six items to give the highest quality scores, and four papers only reported three of the six items, giving the lowest quality scores.

**Table 1:** SYRCLES risk of bias assessment.

**Publication**	**Similar baseline characteristics for each treatment groups**	**Random housing of animalsutilized**	**Blindingof outcome assessor(s)**	**Incomplete outcome dataaddressed**	**Evidence of non-selective outcome reporting s**	**Study free from othersources of bias**
Ai Erken et al. (32)						
Alkarmi et al. (23)						
Araujo et al. (24)						
Barriga et al. (25)						
Chernyakhovskaya et al. (26)						
Chimyshkyan et al. (27)						
Dutta et al. (33)						
Deng et al. (37)						
Faubert et al. (28)						
Hamajima et al. (29)						
Ledingham et al. (35)						
Li et al. (36)						
Liwski et al. (34)						
Svet-Moldavsky et al. (30)						
Szkudlinski et al. (31)						

*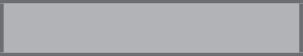 Low risk 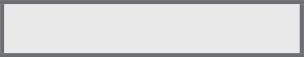 Unclear risk 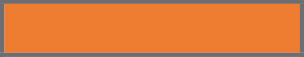 High risk*.

Most of the publications were unclear on their reporting of randomization and blinding. Reports of random housing were unclear in 53% of publications and blinding of outcome assessors was unclear in 40%. These two features were deemed in this review to be of minimal importance for the assessment of allograft rejection. Importantly, 93% of included publications reported similar baseline characteristics, a necessary component for comparing animals in transplantation studies. Additionally, 87% of publications were free from other sources of bias.

### Individual Study Results and Synthesis of Results

**Figure 3:**
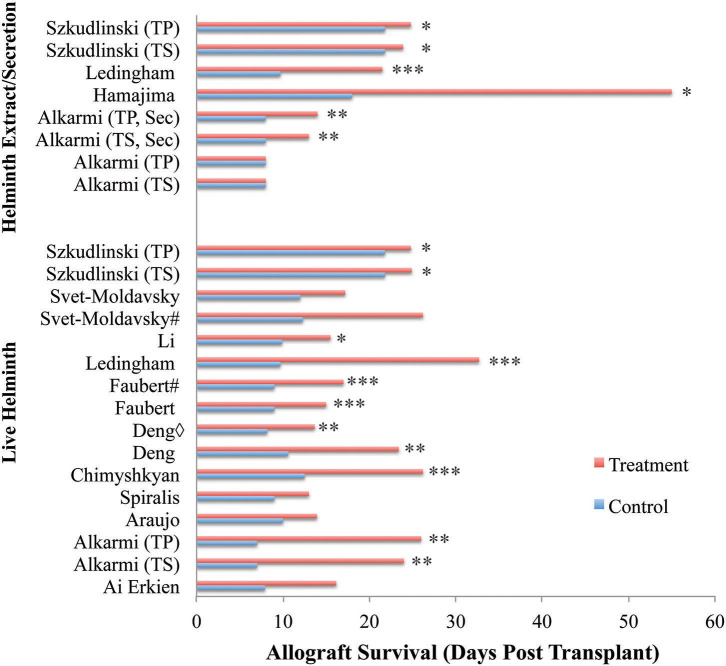
Allograft survival across reviewed publications. Entries show study types differing by treatment type (live vs. helminth extract/secretion), and administration timeframe relative to transplantation. Almost all publications reported significant improvements to allograft survival with the administration of helminth therapy regardless of live or product-based therapeutic forms. Only one publication reported no significant difference to allograft survival with the administration of soluble worm extracts, but had increased survival with all other treatment forms (23). Additionally, two papers did not find significant improvement despite demonstrating considerable trends in allograft survival (26, 30). Three papers were excluded from analysis noting that a clear survival time in days was not provided. Key: ♢ indicates same helminth treatment applied to different transplanted organs. # indicates differing timing of helminth administration. Sec, secretion; TS, *T. spiralis*; TP, *T. pseudospiralis*. *p < 0.05, **p < 0.01, ***p < 0.001.

All 15 publications reported significant improvements to allograft survival with the administration of both live and product-based helminth therapy (Figure 3). Alterations to study design features including, helminth genus/species, parasite burden, and allograft type, showed an impact on the extent of allograft survival. The results of these publications are summarized in Table 2.

**Table 2:** Impacts of study design on allograft survival.

**Study**	**Helminth**	**Allograft model**	**Live infection or product**	**Parasite burden Administration route Life stage**	**Administration time (days)**	**Rejection (days post-transplantation) Controls**	**Rejection (days post-transplantation) Treatment**	***p*-value**
**HELMINTH GENUS: ECHINOCOCCUS**								
Ai Erkien et al. (32)	*Echinococcus multilocularis*	Rat heart	Live	20% larval suspension IP injection Larvae	Not defined	7.92 ± 1.93	16.17 ± 3.19	<0.05
Li et al. (36)	*Echinococcus multilocularis*	Rat liver	Live	20% larval suspension IP injection Larvae	Not defined	9.9 ± 2.3	15.5 ± 3.9	<0.05
**HELMINTH GENUS: SCHISTOSOME**								
Araujo et al. (24)	*Schistosoma mansoni*	Mouse skin	Live	80 cercariae IP injection Cercariae	30 days prior	10	13.93	<0.001
Dutta et al. (33)	*Schistosoma mansoni*	Mouse non-vascularized Heart	Product; recombinant protein	50 μg Lacto-*N-*fucopentaose III SC injection Product	1 day prior and 4 days post-transplant	Not defined	Not defined	<0.003
	*Schistosoma mansoni*	Mouse vascularised Heart	Product; recombinant protein	50 μg Lacto-*N-*fucopentaose III SC injection Product	1 day prior and 4 days post-transplant	Not defined	Not defined	0.008
**HELMINTH GENUS: PARAGONIMUS**								
Hamajima et al. (29)	*Paragonimus westermani*	Mouse skin	Product; soluble worm extract	30 μg/kg IP injection Neutral thiol protease (NTP) from larvae	4 days prior	18 ± 0.5	104 ± 33	<0.05
**HELMINTH GENUS: NIPPOSTRONGYLUS**								
Ledingham et al. (35)	*Nippostrongylus brasiliensis*	Rat kidney	Live	3,500 larvae SC injection Larval	4 days prior	9.7 ± 1.2	32.7 ± 11.3	<0.001
	*Nippostrongylus brasiliensis*	Rat kidney	Product; soluble worm extract	200 worm equivalents SC injection Extract	4 days prior	9.7 ± 1.2	21.5 ± 4.6	<0.001
Liwski et al. (34)	*Nippostrongylus brasiliensis*	Mouse heart	Live	800 larvae SC injection Larval	4 days prior	Between days 9 and 12	Between days 20 and 26	<0.03
**HELMINTH GENUS: TRICHENELLA**								
Alkarmi et al. (23)	*Trichinella spiralis*	Mouse skin	Live	300 larvae Oral inoculation Larval	3 days post-transplant	7	24	<0.01
	*Trichinella pseudospiralis*	Mouse skin	Live	300 larvae Oral inoculation Larval	3 days post-transplant	7	26	<0.01
	*Trichinella spiralis*	Mouse skin	Product; soluble worm extract	50 μg IP injection Extract	Various days post-transplant	8	8	Not stated
	*Trichinella pseudospiralis*	Mouse skin	Product; soluble worm extract	50 μg IP injection Extract	Various days post-transplant	8	8	Not stated
	*Trichinella spiralis*	Mouse skin	Product; native secretions from worm	50 μg IP injection Native secretions	Various days post-transplant	8	13	<0.01
	*Trichinella pseudospiralis*	Mouse skin	Product; native secretions from worm	50 μg IP injection Native secretions	Various days post-transplant	8	14	<0.01
Barriga et al. (25)	*Trichinella spiralis*	Mouse skin	Live	45 larvae Oral inoculation Larval	29 days prior	Between 12 and 18 days	Between 18 and 23 days	Not stated
	*Trichinella spiralis*	Mouse skin	Product; soluble worm extract	0.2 mg TsE protein IP injection Product	29 days prior	Between 12 and 18 days	Between 18 and 23 days	Not stated
Chernyakhovskaya et al. (26)	*Trichinella spiralis*	Mouse skin	Live	70–90 larvae Oral Inoculation Larval	27 days prior	9.5	15	Not stated
Chimyshkyan et al. (27)	*Trichinella spiralis*	Mouse skin	Live	70–90 larvae Oral Inoculation Larval	Not defined	12.5	26.2	<0.001
Deng et al. (37)	*Trichinella spiralis*	Mouse heart	Live	300 larvae Oral Inoculation Larval	28 days prior	10.60 ± 0.75	23.40 ± 1.99	<0.01
	*Trichinella spiralis*	Mouse skin	Live	300 larvae Oral Inoculation Larval	28 days prior	8.17 ± 0.40	13.67 ± 0.56	<0.01
Faubert et al. (28)	*Trichinella spiralis*	Mouse skin	Live	Serum from mice infected with 100 larvae Oral inoculation Serum	Up to 3 days prior	9 ± 0.6	15 ± 0.6	<0.001
	*Trichinella spiralis*	Mouse skin	Live	500 larvae Oral inoculation Larval	30 days prior	9 ± 0.6	17 ± 0.7	<0.001
Svet-Moldavsky et al. (30)	*Trichinella spiralis*	Mouse skin	Live	75–85 larvae Unclear Larval	23 days prior	12.3	26.2	Not stated
	*Trichinella spiralis*	Mouse skin	Live	75–85 larvae Unclear Larval	7 days prior	12.0	17.2	Not stated
Szkudlinski et al. (31)	*Trichinella spiralis*	Mouse skin	Live	80–100 larvae IP injection Larval	23 days prior	21.8 ± 0.5	24.9 ± 0.5	<0.05
	*Trichinella pseudospiralis*	Mouse skin	Live	80–100 larvae IP injection Larval	23 days prior	21.8 ± 0.5	24.8 ± 0.5	<0.05
	*Trichinella spiralis*	Mouse skin	Product; soluble worm extract	Extract isolated from 100 mg larvae SC injection Product	23 days prior	21.8 ± 0.5	23.9 ± 0.7	<0.05
	*Trichinella pseudospiralis*	Mouse skin	Product; soluble worm extract	Extract isolated from 100 mg larvae SC injection Product	23 days prior	21.8 ± 0.5	24.8 ± 0.6	<0.05

### Allograft Survival

Allograft survival was significantly improved in helminth-treated groups when compared to control-treated groups within 12 of the 15 publications, illustrated by reports of marked decreases to necrotic lesions at the allograft site. Only one study noted no improvement to allograft survival with the administration of a soluble worm extract, despite seeing improvements to allograft survival with the administration of infective larvae or secretions from the same worm in the same transplant model (23). Helminth administration of both live and product-based interventions improved allograft survival by an average of 13.6 days across all studies. The minimum improvement to graft survival of 2.1 days was observed with the administration of an extract isolated from 100 mg of *T. spiralis* larvae within a skin graft model (31). The maximum improvement to graft survival was 86 days in a skin graft model involving the administration of 30 μg/kg of neutral thiol protease isolated from *P. westermani* (29).

Improved allograft survival was supported in six studies by supplementary secondary analysis in the form of immunohistochemistry, flow cytometry and/or serum cytokine detection. Using these different analytical approaches revealed a predominant switch in the phenotype of immune response. The prolonged allograft survival mediated by live infection was consistently associated with a significant decrease in the levels of pro-inflammatory cytokines (primarily IFNγ and IL-17) and a parallel increase in Th2/Treg cytokines (IL-4 and IL-10) (34–37). Supporting these observations, a number of studies showed increased numbers of Tregs (Foxp3^+^, CD25^+^) within the graft tissue in response to both helminth infection and administration of helminth-derived products (32, 33, 37). In addition, there were larger quantities of eosinophils in graft tissue of animals receiving helminth therapy (32, 33).

### Impacts of Study Design

#### Helminth Genus and Species

Within two studies of skin allograft transplantation, administration of live *T. spiralis* and *T. pseudospiralis* larvae produced similar improvements to allograft survival in comparison to control (23, 31), suggesting that alteration of the parasite species does not impact the positive outcome. In contrast, changing the helminth genus resulted in altered impacts to allograft survival. Across two studies with similar study design (administering 70–90 larvae within a skin allograft model), a change from the genus *Schistosoma* to *Trichinella* vastly improved allograft survival from 13.93 to 26.2 days (24, 27). This effect was also seen in two different studies where they compared the administration of *Echinococcus* or *Trichinella* within heart allograft models, with allograft survival extending from 16.17 to 23.4 days, respectively (32, 37).

#### Chronicity and Worm Burden of Helminth Infection

The duration of the parasite infections appeared to influence the outcome of allograft survival. Administration of 75–85 *T. spiralis* larvae 23 days prior to the transplantation of a skin graft elicited greater graft survival (26.2 days) than larvae given 7 days before (17.2 days) (30). As helminths do not replicate in their hosts, but rather produce eggs for transmission, this difference in outcome cannot be attributed to an increase in parasite numbers over time. However, increasing the worm burden does have a positive impact on graft survival. Increasing the infective dose of *T. spiralis* or *T. pseudospiralis* to 300 larvae resulted in the same improvement in allograft survival (24 and 26 days) (23) as the long-term infection, despite being delivered 3 days post-transplant. The importance of worm burden is further supported in rat models of solid organ transplant, where the administration of 800 larvae of *N. brasiliensis* 4 days prior to a heart transplant resulted in allograft survival to 20–26 days, compared to 9–12 days for the controls (34). Increasing the infective burden to 3,500 larvae further extended the survival of a solid organ (kidney) allograft to 32.7 days (35).

#### Therapeutic Differences Between Live and Product-Based Helminth Therapies

Of the live infection therapies, administration of *N. brasiliensis* showed the most significant prolongation of graft survival (34). However, overall, across all studies the largest increase to allograft survival was seen with the administration of a recombinant protein derived from *P. westermani*, which showed an extension of allograft survival 86 days beyond that seen in the control animals (control: 18 ± 0.5, treatment: 104 ± 33, *p* <0.05) (29).

A direct comparison between live infection and the administration of helminth-derived products was specifically investigated within four separate studies (23, 25, 31, 35). Each publication maintained identical helminth genus or species, only differing in the type of product and administration route. Within a skin graft model, the administration of 45 live *T. spiralis* larvae by the natural route of infection (oral inoculation) induced no difference in graft survival when compared to that induced by a single IP injection of 0.2 mg of a soluble protein extract of the same parasite (25). Both prolonged allograft survival to between 18 and 23 days in comparison to the 12–18 days seen with control (25). Similar results were also achieved within another skin allograft model, in which either *T. spiralis* or *T. pseudospiralis* was administered as either a live infection (80–100 larvae orally) or a soluble worm extract (prepared from 100 mg larvae) (31). Differences in graft survival prolongation were minimal for both species: *T. spiralis* (live: 24.9 ± 0.5 days, extract: 23.9 ± 0.7 days); *T. pseudospiralis* (live: 24.8 ± 0.5, extract: 24.8 ± 0.6 days, control: 21.8 ± 0.5). In contrast, another skin graft study which tested the same parasite species showed that live infection of either species (300 larvae delivered orally) resulted in extended allograft survival to 24 days whilst the IP administration of soluble worm extract (50 μg) did not significantly extend graft survival past that of the control group (day 8) (23). Thus, in the application of *Trichinella* species, the administration of low quantities (50 μg) of either native secretions or a soluble extract of the worm were less effective or ineffective, respectively, in preventing the rejection of skin grafts compared to studies where either higher doses (designed to imitate the presence of live worms) or live infection was used. This observation was supported by a study in a kidney allograft model (35), where the administration of 3,500 *N. brasiliensis* larvae markedly improved allograft survival to 32.7 ± 11.3 days, whilst the administration of a soluble worm extract collected from 200 larvae only increased graft survival to 21.5 ± 4.6 days (control: 9.7 ± 1.2 days, *p* <0.001).

#### Allograft Type

Differences in the type of allograft model used also impacted on the extent of allograft survival. The smallest improvement to allograft survival was seen with skin allografts. This effect was well-modeled within one investigation of the effects of 300 *T. spiralis* larvae on both heart and skin allografts (37). Whilst the allograft survival of both transplant types was prolonged with helminth administration when compared to control, the effect in the heart model was greater, with graft survival extended to 23.4 days in comparison to 10.6 days for control (*p* <0.01). Within the skin model, allograft survival was limited to 13.67 days in comparison to control (*p* <0.01). The graft survival duration for other solid organ (heart and liver) transplants were similarly impacted by helminth infection. Two studies investigating the effect of *E. multilocularis* live therapy on both liver and heart allografts (32, 36) were matched according to administration and dosing. Both liver (15.5 days, *p* <0.05) and heart (16.17 days, *p* <0.05) allografts survived to a similar extent in each study. These comparisons stand as evidence for the differences in allograft survival being influenced by the type of tissue being transplanted.

## Discussion

### Summary of Evidence

This systematic review has determined that in all 15 publications allograft survival was significantly improved in helminth-treated groups, with all studies reporting marked decrease in graft necrosis. The positive impacts of helminth therapy on allograft survival were clearly seen across multiple animal models of allogeneic transplantation. The finding that both live infection and the administration of helminth-derived products were both effective at preventing allograft rejection, is consistent with a previous literature review of this topic (38) and suggests that parasite secreted molecules can be as efficacious as live infection. This similarity in outcomes could be attributed (with the support of data presented in six of the fifteen publications) to a common mechanism of immune modulation. Both parasite infection and the administration of a recombinant version of a parasite-derived protein resulted in a decreased pro-inflammatory immune response concomitant with a rise in Th2/Treg immune response; a profile of immunity known to support the prolonged survival of a transplanted graft (18).

The effectiveness of the helminth therapy evaluated in the 15 publications appeared to be dependent on the type of tissue that was transplanted. This was most strikingly evident in a comparison between the survival of a skin and heart allograft after infection with *T. spiralis*, with the heart surviving 10 days longer than the skin graft (37). It has long been noted that skin grafts are more readily rejected than heart transplants (39). Skin grafts are considered to be more potent stimulators of the immune system as they contain large numbers of Langerhans cells, a population of cells with excellent antigen presentation ability (40). In addition, it has been demonstrated that the slow nature of developing vasculature in skin grafts (days for skin grafts vs. hours for heart grafts) results in prolonged ischemic injury which, in turn, stimulates inflammatory signals that increase T cell priming and migration to the site of transplantation (41). It has also been reported that simply the difference in size of the transplanted tissue influences the rate of rejection, as larger number of effector T cells are needed for the destruction of a donor heart in comparison to a skin graft (42). While the immune response in the parasite infected recipients of heart and skin grafts was not compared, it could be hypothesized that the modulation of immune responses in this instance by the parasite was simply not sufficient to counteract the acute and potent inflammation driven by the skin graft.

Two elements of a helminth infection dictate the profile and potency of the host's immune response; time and worm burden. For some parasites, the development of the characteristic predominance of Th2/Treg immune response takes time as it is often dependent on the maturation of the parasite, the deposition of eggs, or simply the time it takes to migrate to the anatomical location in which they ultimately reside (43–45). In addition, it has been reported that individuals harboring greater worm numbers display a stronger regulatory response (46). In the skin and heart transplant studies mice were infected with *T. spiralis* 28 days prior to transplantation (23, 37). For this particular parasite, a Th2 immune response is immediately induced after infection in mice, developing into a mixed Th17/Th2 response in the proceeding weeks post-infection, followed by a predominating Treg population (47, 48). This would suggest that the presence of a regulatory T cell population at the time of transplantation, which although effective against the heart transplant, did not impact the immune response to a great enough extent for the skin graft. Such a notion could be supported by the observation that the infection of mice with the same dose (300 larvae) of *T. spiralis* 3 days after a skin transplantation resulted in the survival of the graft for 24 days, compared to 13 days when the parasite was administered 28 days before transplantation (23, 37). Although not examined, perhaps the nature of the immediate Th2 immune response induced by the infection with the parasite around the time of transplantation was more appropriate to regulate the inflammation induced by the skin graft.

The importance of inducing the correct immune response to facilitate transplant survival is also illustrated when comparing the outcomes of infection with different genera of parasites. On the surface it appears that infection with live *Trichinella* species are far more effective in preventing skin transplant rejection compared to *S. mansoni* (24, 27). However, in contrast to the delivery of *Trichinella* larvae, *S. mansoni* was not administered via its natural route of infection (skin), which likely impacted on the ability of the parasite to fully modulate the immune response as expected.

Understanding the biology of the parasite to better exploit its immune modulating power becomes even more critical in the selection and analysis of the efficacy of helminth-derived products. This is most evidently demonstrated in a skin graft study that compared the impact of live infection with either *T. spiralis* or *T. pseudospiralis* to the IP administration of soluble extracts of worms (50 μg) and to an IP injection of the native secretions of the parasite (50 μg) (23). It is assumed that the immune modulators produced by helminth parasites are typically found within their secreted products, as these are the compounds most likely to directly interact with the host immune cells (15). In contrast, experimentally prepared extracts contain the complete soluble protein content from homogenized worms, some of which are originally produced to function intracellularly within the parasite and not in the external environment of the host tissue. Reflecting this adaptation of biological function, the IP administration of the native secretions extended the allograft survival to 14 days in this model (23). Although this is less effective than oral infection (24–26 days), it was a significant (*p* <0.01) improvement compared to the same dose of soluble extracts prepared by either homogenizing the worms (8 days) or the control group (7 days). To properly compare the native secretions of the parasite to the live infection, a dose which more accurately represents the quantity of proteins secreted by an equivalent number of larvae should be administered to the transplant recipient.

An alternative explanation for the reduced efficacy of the native secretions compared to live infection is that the continued presence of the parasite may be necessary to elicit the regulatory immune response in the host (7, 49). However, this seems less likely when the results from the two studies that utilized isolated helminth-derived products are evaluated. Only two injections (50 μg) of a recombinantly produced version of the *S. mansoni* pentasaccharide LNFP-III were required to extend median graft survival of heart transplant compared to controls (33). This molecule is found on the surface of the eggs of *S. mansoni*, the production of which coincides with the development of a potent Th2/Treg immune response in this parasite infection (43). The positive impact on the heart transplant was mediated by the differentiation of macrophages toward an M2 phenotype, which subsequently promoted the accumulation of Tregs in the draining lymph nodes of the donor organs. Adoptive transfer of these LNFP-III activated macrophages was sufficient to also significantly prolong allograft survival (33). Perhaps even more effective was the native neutral-thiol protease harvested from the secretions of *P. westermani*, as only a single IP injection was required to provide the greatest protection to the graft across all of the studies examined, extending the survival of a skin graft to 86 days beyond the control animals (29). Furthermore, the dose of protein delivered (30 μg/kg) was far lower than any of the other experiments that described the effect of soluble extracts or secreted products (50–200 μg per mouse). This protein is naturally secreted by the parasite and acts as a cysteine protease with a broad substrate specificity supporting the cleavage of a broad range of host proteins (50) suggesting an ability to interfere with the host immune signaling proteins and possibly contribute to the potent effect observed in this transplant model.

### Limitations

The review was restricted by the lack of immunological evidence presented in each paper. Of the 15 publications evaluated, nine provided the primary outcome of allograft survival with no additional supporting evidence of immune profiling. This placed limitations on the conclusions that could be drawn, meaning that similarities and differences between therapies on allograft survival could only be assumed based on our current knowledge of transplant and parasite immunology. Additionally, due to the heterogeneous nature of all publications meta-analysis could not be performed, and thus a subjective qualitative synthesis was used for analysis. To reduce the subjective nature of this analysis, input from multiple reviewers was gathered when performing the narrative synthesis. Despite this, lack of statistical evidence does preclude the application of this review to influence the development of helminth-based therapies for enhanced allograft survival. In future, further publications with similar study designs may allow for meta-analysis to validate the conclusions found in this review.

## Conclusions

**Figure 4:**
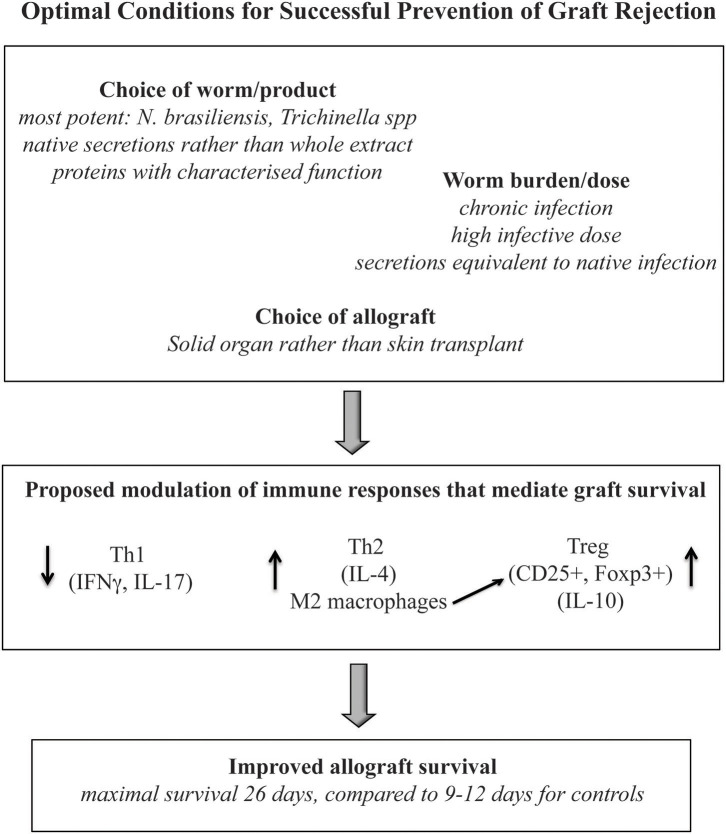
A summary of the immune profile, parasite and graft type which reported optimal improvements to allograft survival.

Despite the limitations, this review provides comparative evaluation of the effects of helminth therapy on allograft survival across multiple models of allogeneic transplantation. While variations in efficacy were most notably linked to the timing and dose of helminth therapy, and to the choice of allograft model, it was clear that all publications reported improvements to allograft survival with the administration of both live and product-based helminth therapy. It is evident that a better understanding of the immune changes induced by the parasites and their products will be essential to developing the most effective therapeutic strategy for specific allografts. Furthermore, to elucidate which parasite-derived molecules are likely to be most effective, a more appropriate analysis of these compounds is required, with consideration for their known or predicted biological function in the host. Nonetheless, the evidence presented here supports the proposition that helminth therapy represents a viable treatment form of immunosuppressive therapy. By careful selection of the helminths or helminth products that are best suited to particular allograft types and consideration of the required dose/chronicity to induce the most appropriate immune response, an optimal protocol can be achieved to provide the most significant immunomodulation to promote successful allograft survival (Figure 4).

## Data Availability Statement

All datasets presented in this study are included in the article/Supplementary Material.

## Author Contributions

WH conceived the idea. MK searched the literature and drafted the manuscript. WH, HB, and SD commented on the structure of manuscript, provided the critical intellectual input, and edited the manuscript. All authors read and approved the final manuscript.

## Conflict of Interest

The authors declare that the research was conducted in the absence of any commercial or financial relationships that could be construed as a potential conflict of interest.

## Supplementary Material

Supplementary Table 1 Study design characteristics.

## Abbreviations

ELISA: enzyme-linked immunosorbent assay
FACS: Fluorescence-activated cell sorting
IP: intra-peritoneal
LNFP-III: Lacto-*N*-fucopentaose III
NTP: Neutral thiol protease
PCR: polymerase chain reaction
SC: subcutaneous
TsE: *Trichinella spiralis* extract.
