# Supplementary material for: Effectiveness of Helminth Therapy in the Prevention of Allograft Rejection: A Systematic Review of Allogeneic Transplantation
Source: Front Immunol. 2020 Aug 7;11:1604. doi: 10.3389/fimmu.2020.01604 (PMC7426368; doi:10.3389/fimmu.2020.01604)
Supplement: Supplementary Table 1 — Study design characteristics. [file Table_1.pdf]

Supplementary Table 1

Study	Helminth	Allograft model	Live infection or product	Parasite burden<br>Administration route<br>Time of administration<br>Life stage	Sample Size	Primary Outcome	Secondary Measurement
Helminth Genus: Echinococcus							
Ai Erkien et al. (2012) (32)	*Echinococcus multilocularis*	Rat heart	Live	20% larval suspension<br>IP injection<br>Not defined<br>Larvae	12	Graft survival; cessation of cardiac beating	1. Histopathology<br>2. Immunohistochemistry<br>3. Flow cytometry
Li et al. (2011) (36)	*Echinococcus multilocularis*	Rat liver	Live	20% larval suspension<br>IP injection<br>Not defined<br>Larvae	16	Graft survival; necrosis	1. Histopathology<br>2. Flow cytometry<br>3. PCR<br>4. ELISA
Helminth Genus: Schistosoma							
Araujo et al. (1977) (24)	*Schistosoma mansoni*	Mouse skin	Live	80 cercariae<br>IP injection<br>30 days prior<br>Cercariae	25	Graft survival; bleeding and shrinking of graft and detachment of border noted as rejection	None
Dutta et al. (2010) (33)	*Schistosoma mansoni*	Mouse non-vascularised Heart	Product; recombinant protein	50µg Lacto-*N*-fucopentaose III<br>SC injection<br>1 day prior and 4 days post-transplant<br>Product	10	Graft survival; cessation of cardiac beating	1. Flow cytometry
	Schistosoma mansoni	Mouse vascularised Heart	Product; recombinant protein	50µg Lacto-*N*-fucopentaose III<br>SC injection<br>1 day prior and 4 days post-transplant<br>Product	5	Graft survival; cessation of cardiac beating	1. Flow cytometry
Helminth Genus: Paragonimus							
Hamajima et al. (1994) (29)	*Paragonimus westermani*	Mouse skin	Product; soluble worm extract	30µg/ kg<br>IP injection<br>4 days prior<br>Neutral thiol protease (NTP) from larvae	6	Graft survival; rejection classified as 85% of more induration and no hair growth	None
Helminth Genus: Nippostronglyus							
Ledingham et	*Nippostrongylus*	Rat kidney	Live	3500 larvae	Not	Graft survival;	1. Histopathology

al. (1996) (35)	*brasiliensis*			SC injection<br>4 days prior<br>Larval	defined	increasing signs of morbidity due to kidney failure	2. Immunohistochemistry<br>3. Flow cytometry
	Nippostrongylus brasiliensis	Rat kidney	Product; soluble worm extract	200 worm equivalents<br>SC injection<br>4 days prior<br>Extract	6	Graft survival; increasing signs of morbidity due to kidney failure	1. Histopathology<br>2. Immunohistochemistry<br>3. Flow cytometry
Liwski et al. (2000) (34)	*Nippostrongylus brasiliensis*	Mouse heart	Live	800 larvae<br>SC injection<br>4 days prior<br>Larval	5	Graft survival; heart function	1. ELISA – cytokines<br>2. FACS<br>3. Intracellular IL-4<br>4. Cytotoxic T cell activity
Helminth Genus: Trichinella							
Alkarmi et al. (1995)(23)	*Trichinella spiralis*	Mouse skin	Live	300 larvae<br>Oral inoculation<br>3 days post-transplant<br>Larval	50	Graft survival; detachment of graft	None
	Trichinella pseudospiralis	Mouse skin	Live	300 larvae<br>Oral inoculation<br>3 days post-transplant<br>Larval	50	Graft survival; detachment of graft	None
	Trichinella spiralis	Mouse skin	Product; soluble worm extract	50μg<br>IP injection<br>Various days post-transplant<br>Extract	10	Graft survival; detachment of graft	None
	Trichinella pseudospiralis	Mouse skin	Product; soluble worm extract	50μg<br>IP injection<br>Various days post-transplant<br>Extract	10	Graft survival; detachment of graft	None
	Trichinella spiralis	Mouse skin	Product; native secretions from worm	50μg<br>IP injection<br>Various days post-transplant<br>Native secretions	10	Graft survival; detachment of graft	None
	Trichinella pseudospiralis	Mouse skin	Product; native secretions from worm	50μg<br>IP injection<br>Various days post-transplant<br>Native secretions	10	Graft survival; detachment of graft	None

Barriga et al. (1978) (25)	*Trichinella spiralis*	Mouse skin	Live	45 larvae<br>Oral inoculation<br>29 days prior<br>Larval	4	Graft survival;<br>1x – mild inflammation<br>2x – intense inflammation<br>3x – necrosis<br>4x – sloughing	None
	Trichinella spiralis	Mouse skin	Product; soluble worm extract	0.2mg TsE protein<br>IP injection<br>29 days prior<br>Product	4	Graft survival;<br>1x – mild inflammation<br>2x – intense inflammation<br>3x – necrosis<br>4x – sloughing	None
Chernyakhovskaya et al (1972) (26)	*Trichinella spiralis*	Mouse skin	Live	70-90 larvae<br>Oral Inoculation<br>27 days prior<br>Larval	N/A	Graft survival; rejection noted as oedema and haemorrhages on the surface of the graft. Complete necrosis noted as destruction of graft epithelium and appearing scar	None
Chimyshkyan et al. (1976) (27)	*Trichinella spiralis*	Mouse skin	Live	70-90 larvae<br>Oral Inoculation<br>Not defined<br>Larval	N/A	Graft survival; necrosis	None
Deng et al. (2016) (37)	*Trichinella spiralis*	Mouse heart	Live	300 larvae<br>Oral Inoculation<br>28 days prior<br>Larval	5	Graft survival; rejection classified as cessation of cardiac beating	1. Histopathology<br>2. Flow cytometry<br>3. Luminex - cytokines
	Trichinella spiralis	Mouse skin	Live	300 larvae<br>Oral Inoculation<br>28 days prior<br>Larval	5	Graft survival; rejection defined as >80 % necrosis of the transplanted skin surface as well as the appearance of desiccation and shrinkage.	1. Histopathology<br>2. Flow cytometry<br>3. Luminex - cytokines
Faubert et al. (1975) (28)	*Trichinella spiralis*	Mouse skin	Live	Serum from mice infected with 100 larvae<br>Oral inoculation<br>Up to 3 days prior<br>Serum	10	Graft survival; oedema, necrosis	None

	Trichinella spiralis	Mouse skin	Live	500 larvae<br>Oral inoculation<br>30 days prior<br>Larval	15	Graft survival; oedema, necrosis	None
Svet-Moldavsky et al. (1969) (30)	*Trichinella spiralis*	Mouse skin	Live	75-85 larvae<br>Unclear<br>23 days prior<br>Larval	9	Graft survival; oedema, necrosis	None
	Trichinella spiralis	Mouse skin	Live	75-85 larvae<br>Unclear<br>7 days prior<br>Larval	8	Graft survival; necrosis	None
Szkudlinski et al. (1997) (31)	*Trichinella spiralis*	Mouse skin	Live	80-100 larvae<br>IP injection<br>23 days prior<br>Larval	12	Graft survival; percentage of area of necrosis	None
	Trichinella pseudospiralis	Mouse skin	Live	80-100 larvae<br>IP injection<br>23 days prior<br>Larval	12	Graft survival; percentage of area of necrosis	None
	Trichinella spiralis	Mouse skin	Product; soluble worm extract	Extract isolated from 100mg larvae<br>SC injection<br>23 days prior<br>Product	12	Graft survival; percentage of area of necrosis	None
	Trichinella pseudospiralis	Mouse skin	Product; soluble worm extract	Extract isolated from 100mg larvae<br>SC injection<br>23 days prior<br>Product	12	Graft survival; percentage of area of necrosis	None
